# Improving the Robustness of Human-Machine Interactive Control for Myoelectric Prosthetic Hand During Arm Position Changing

**DOI:** 10.3389/fnbot.2022.853773

**Published:** 2022-06-07

**Authors:** Ang Ke, Jian Huang, Jing Wang, Jiping He

**Affiliations:** ^1^Key Laboratory of Ministry of Education for Image Processing and Intelligent Control, School of Artificial Intelligence and Automation, Huazhong University of Science and Technology, Wuhan, China; ^2^Shenzhen Huazhong University of Science and Technology Research Institute, Shenzhen, China; ^3^Department of Intelligent Robots and Systems, Beijing Institute of Technology, Beijing, China

**Keywords:** gesture recognition, arm movement, EMG-FMG control, post-processing, robustness

## Abstract

Robust classification of natural hand grasp type based on electromyography (EMG) still has some shortcomings in the practical prosthetic hand control, owing to the influence of dynamic arm position changing during hand actions. This study provided a framework for robust hand grasp type classification during dynamic arm position changes, improving both the “hardware” and “algorithm” components. In the hardware aspect, co-located synchronous EMG and force myography (FMG) signals are adopted as the multi-modal strategy. In the algorithm aspect, a sequential decision algorithm is proposed by combining the RNN-based deep learning model with a knowledge-based post-processing model. Experimental results showed that the classification accuracy of multi-modal EMG-FMG signals was increased by more than 10% compared with the EMG-only signal. Moreover, the classification accuracy of the proposed sequential decision algorithm improved the accuracy by more than 4% compared with other baseline models when using both EMG and FMG signals.

## 1. Introduction

Currently, the most widely used method for the control of an external powered prosthetic hand is the EMG pattern recognition (PR) based control method (Iqbal and Subramaniam, 2018; Parajuli et al., 2019; Yao et al., 2021). Although the performance of the EMG-PR-based control method has been reported to achieve extremely high accuracy academically, the practical application in prosthetic hands is still insufficient. This academical-practical gap comes from several reasons:

The first reason is the accurate classification of taxonomically close motion classes (TCMC) with EMG signals. Many existing academical studies used the taxonomically distant motion classes (TDMC) (Shahzad et al., 2020) to evaluate the performance of PR system, such as hand close/open, wrist flexion/extension. This TDMC partly contributed to the high accuracy of the PR system in academic research, whereas the TDMC is counter-intuitive and unnatural for prosthetic hand control. Compared with the TDMC, the TCMC (such as the grasp types in human grasp taxonomy; Feix et al., 2015) is much more intuitive, but the classification of TCMC is more challenging for EMG-PR-based control.

For this aspect of the academical-practical gap, one of the most influential studies is the open Ninapro databases for naturally-controlled robotic hand prostheses provided by Atzori et al. (2014). Among these databases, the second database (DB2) of Ninapro is the closest to daily life application, which consists of 50 gestures, including the grasping and functional movements of daily-life objects, finger movements, and wrist movements collected from 40 healthy subjects with 12 wireless electrodes. This open database greatly facilitates the decoding of hand movement from EMG since many researchers developed and tested their algorithms on this open database. Among these algorithms, deep learning (DL) is the most commonly used method (Rim et al., 2020; Buongiorno et al., 2021; Rajapriya et al., 2021; Xiong et al., 2021). Atzori et al. (2016) proposed a simple convolutional neural network (CNN) consisting of four convolutional layers. The average accuracy tested on DB2 is only 60.27%. Considering the sequential nature of the EMG signal, Hu et al. proposed an attention-based hybrid CNN and recurrent neural networks (RNN). The accuracy tested on Ninapro DB2 is up to 82.2%. Ding et al. proposed a parallel multiple-scale convolution architecture, which consisted of two parallel blocks for feature extraction. The average accuracy tested on NinaPro DB2 was 78.86%. Wei et al. proposed a multi-view CNN framework, which combined the classical EMG feature sets with a CNN classifier. The average accuracy tested on DB2 is up to 83.7%. Rahimian et al. (2021) proposed a “Few-Shot Learning” framework based on meta-learning. The algorithm achieved an accuracy of 85.94% on new repetitions when tested on Ninapro DB2.

Although these works achieved promising performance on new repetitions (the tested data is a never-seen-before repetition), the training strategy was still different from real-time prosthetic control. The training sessions should always precede the testing ones in temporal coherence in a real-world PR-based control system of hand prosthesis. However, the mentioned works above all failed to (usually the repetitions 1, 3, 4, and 6 in Ninapro DB2 are used for training, and the other two repetitions were used for testing) meet this requirement.

The second reason is the change of surface EMG signal in a real-world environment in contrast to the well-controlled laboratory conditions. This reason is much more challenging than the first aspect. There are many disturbing factors in the real world, such as the electrodes shift, arm position change, electrode-skin impedance, and muscle fatigue (Kyranou et al., 2018; Jung et al., 2021). Among these disturbing factors, dynamically changed arm position is one of the inevitable factors that will degrade the performance of EMG-PR algorithms severely (Radmand et al., 2014; Shin et al., 2016; Teh and Hargrove, 2020). For the real-world application of myoelectric prosthesis, the arm's position will inevitably change when the amputee performs activities of daily living. Hence, it is challenging to train a motion classifier to maintain high performance while moving the arm to different positions, such as the hand grasp gesture classification during a commonly used reach-grasp-moving-release-retract (RGMRR) task.

Several methods have been put forward to minimize the influence of arm position changes in past studies, such as the multi-position classifier (Geng et al., 2012), cascade classifier (Geng et al., 2017), dynamic training (Shahzad et al., 2019), position-invariant features (Asogbon et al., 2020), and other classification algorithms. Multi-modal signals are usually needed for many methods, such as the accelerometry (ACC) signal, the near-infrared spectroscopy (NIRS) (Guo et al., 2015, 2017), electroencephalography (EEG) (Leeb et al., 2011), FMG (Ferigo et al., 2017; Prakash et al., 2020a,b; Huang et al., 2021), and some industry sensors used for human-centered robotic systems (Huang et al., 2020; Yan et al., 2021). Among these signals, the ACC is the most commonly used signal for the complementary of EMG (Geng et al., 2012; Huang et al., 2015; Shahzad et al., 2019), since the tri-axis ACC signal could provide information about arm position. Moreover, the ACC and EMG signal can easily be collected together by a commercial product (such as the Delsys Trigno Wireless System). However, the ACC signal is very sensitive to unwanted motions. Thus both of the multi-position classifier and cascade classifier are suitable for several stationary arm positions.

Similar to EMG, the FMG signal is another good choice that can be used for real-time control (Belyea et al., 2019; Choi et al., 2021), which measures the shape and stiffness change of the muscle during muscle contraction *via* the force sensor. The FMG signal has also been studied for mitigating the influence of limb position. In Ferigo et al. (2017), the author studied the influence of limb position on FMG-PR based natural control of a prosthetic hand, the results showed the classification accuracy was more than 99% in a stationary position. When the arm position was dynamically changed, the classification accuracy degraded significantly. However, the robustness of FMG is still higher than the EMG, whereas the dynamic non-ideal effects of FMG are worse than the EMG due to the working principles of FMG. It is easy to think that combining the EMG signal with the FMG signal may be a good choice to mitigate the influence of dynamic arm position change on PR-based prosthetic hand control. Although there were some studies on gesture recognition based on combined EMG-FMG signals (Connan et al., 2016; Jiang et al., 2020; Ke et al., 2020; Choi et al., 2021), the ability of EMG-FMG sensor for hand grasp types when the arm position is dynamically changing has not been investigated, especially the synergy effects of co-located EMG and FMG signal on the classification of natural hand grasp types in ADLs.

**Figure 1:**
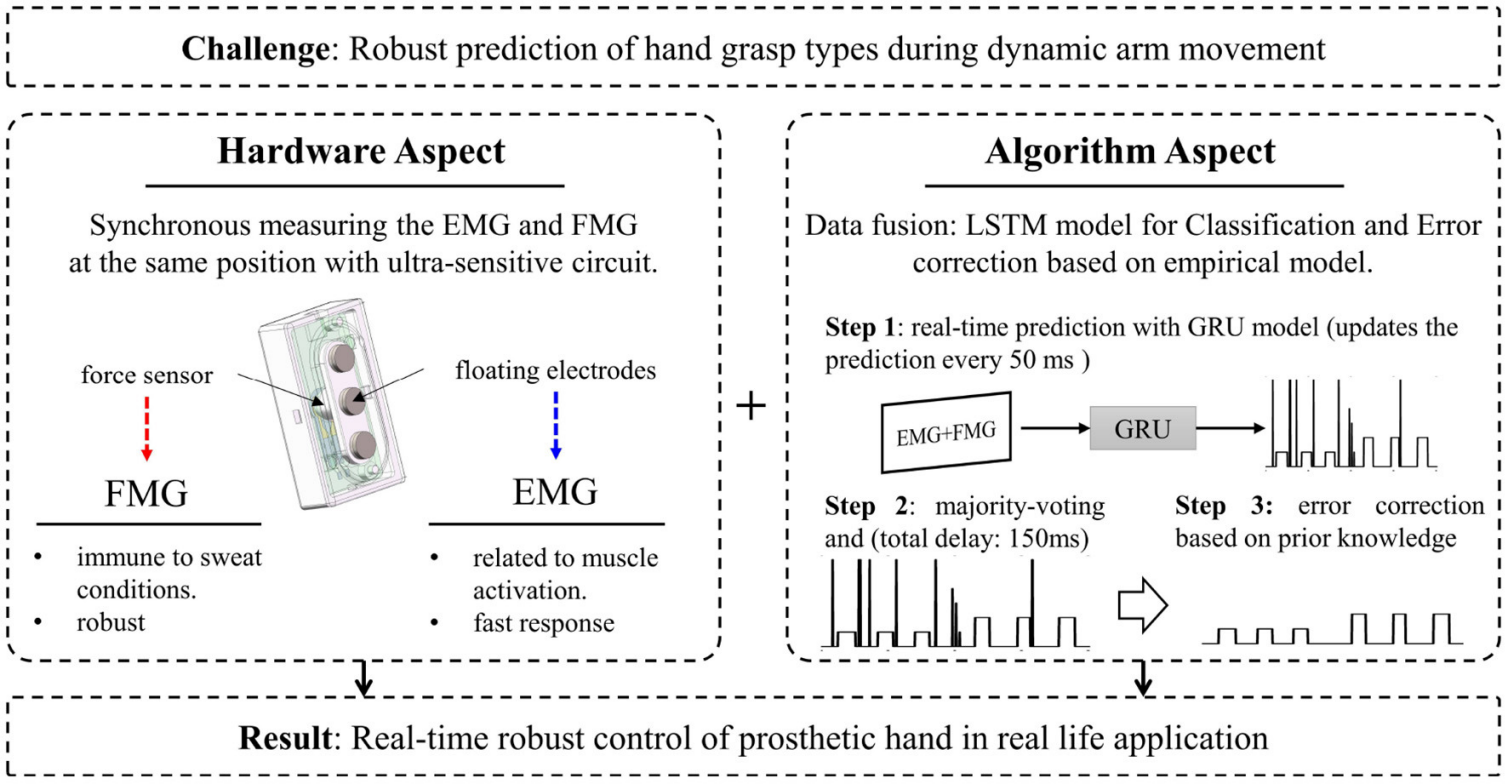
A summary of the approach for robust hand gesture recognition during dynamic arm movement in this study.

In this study, we aimed to put forward a framework to address the problem of intuitively and naturally control of prosthetic hands during dynamic arm position changes. The framework consists of two parts: the hardware and algorithm, as illustrated in Figure 1. On the hardware aspect, the combination of co-located FMG and EMG signals are adopted as the multi-modal strategy, in which the EMG and FMG signal are measured at the same place. On the algorithm side, a sequential decision algorithm that can be used for real-time classification is proposed by combining the RNN-based deep learning model with a knowledge-based post-processing model. The proposed framework was tested on an experiment of the RGMRR task, in which six most commonly used grasp types selected from human grasp taxonomy were used for the training and testing motion classes.

Generally speaking, the main contributions of this study can be summarized as follows:

1. First, we proposed a framework to improve the robustness of intuitively and naturally control of myoelectric prosthetic hands during arm position changing, which considering both the multi-mode signal input solution and the signal fusion algorithm
2. Second, a sequential decision algorithm that can be used for real-time classification is proposed by combining the RNN-based deep learning model with a knowledge-based post-processing model.

## 2. Materials and Methods

### 2.1. Subjects

In this research, eight healthy right-handed volunteers were recruited for our experiment (five males, three females). All subjects were informed about the protocol and risks before the experiment and signed an informed consent form. The experiment was approved by the Ethics Commission of the Yangxin People's Hospital, and it was conducted according to the principles expressed in the Declaration of Helsinki.

### 2.2. Experimental Protocol

**Figure 2:**
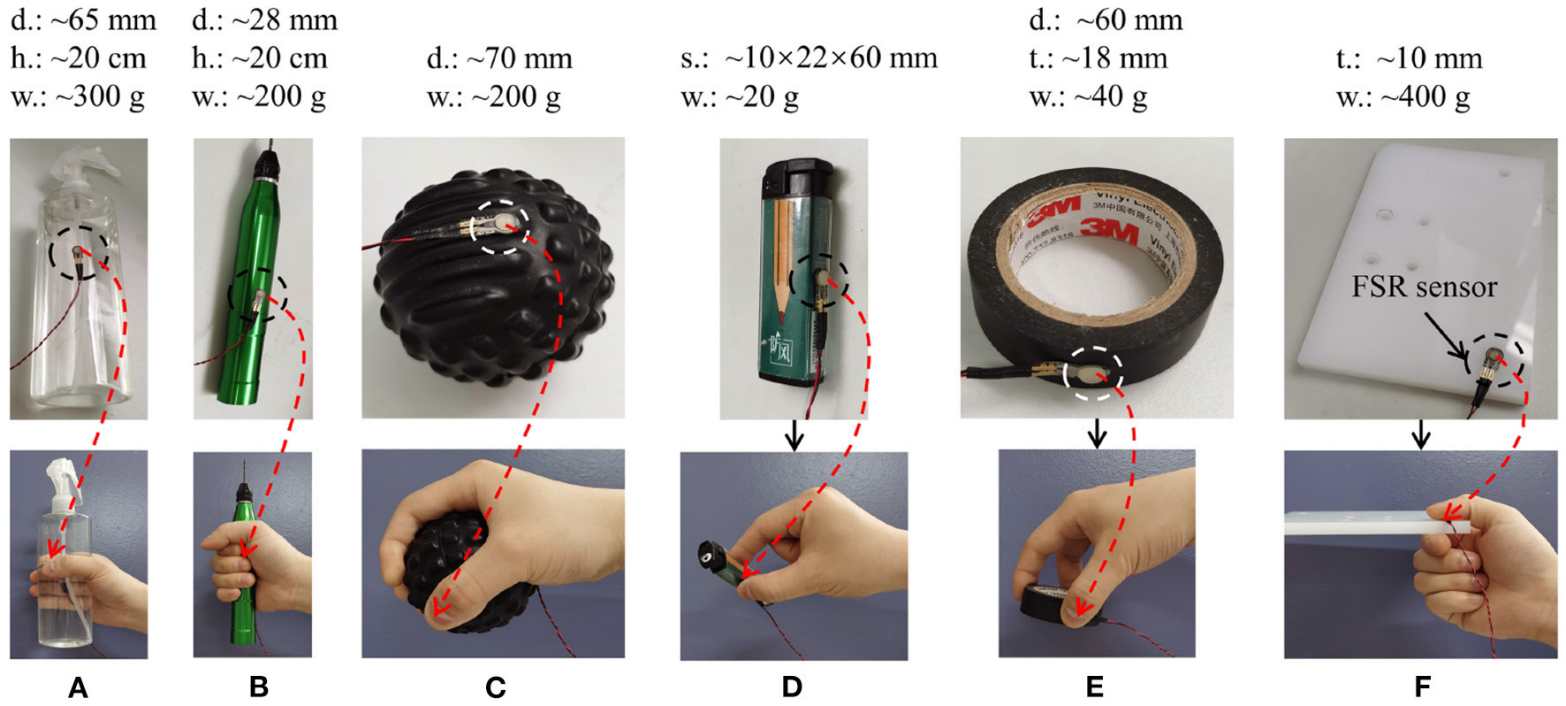
The six grasp types and the corresponding objects used for RGLRR task, they were: **(A)** large diameter cylindrical grasp; **(B)** small diameter cylindrical grasp; **(C)** power sphere grasp; **(D)** tip pinch; **(E)** tripod grasp; **(F)** lateral grasp.

Six natural grasp types selected from the human grasp taxonomy (Feix et al., 2015) were used for the RGMRR task; they were: large diameter cylindrical grasp (LDC), small diameter cylindrical grasp (SDC), power sphere grasp (POS), tip pinch (PIN), tripod grasp (TRI), and lateral grasp (LAT). Six daily-life used objects were selected to execute the corresponding grasps: a plastic bottle containing water, an electric metal drill, an ethylene-vinyl acetate massage ball, a plastic lighter, a roll of electrical tape, and an Alec plate. The size and weight about these objects is shown in Figure 2. A thin-film force-sensing resistor (FSR) was attached to the surface of each object in a feasible position where the fingertip of the thumb contacts the object during grasp (as shown in Figure 2). All subjects were carefully instructed to grasp the objects with their thumb, or index finger pressed on the FSR. With this method, the exact time of grasp can be determined from the output signal of the FSR.

**Figure 3:**
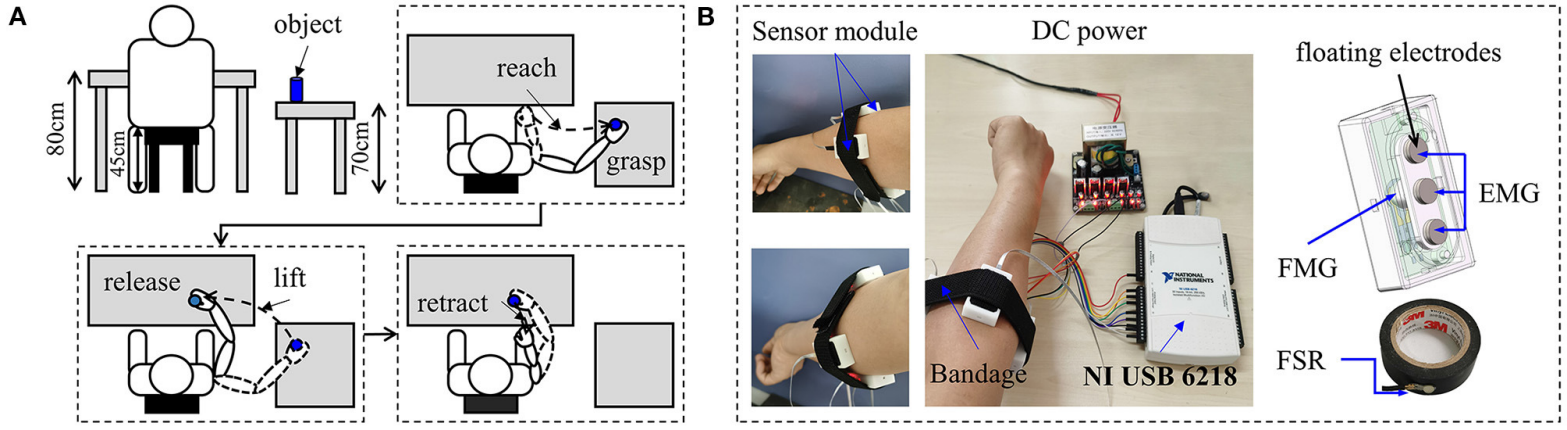
Illustration of the experimental protocol **(A)** and data acquisition method **(B)**.

The layout of the experimental setup and the action of the arm is shown in Figure 3A. The experiment procedure was a repetitive reaching, grasping, moving, releasing, and retracting cycle. In short, the task is to pick up an object from one place and put it in another place (without walking). During the first cycle, the subject was seated comfortably with their hands resting on the knee or naturally hanging down, and this arm position was called relax the arm (AR). Then the subject was asked to raise their arm to reach the object, and this reaching phase is called preparation of grasp (GP). Once the subject grasped the object (GO) firmly, he/she should keep the grasping and then move the object (MO) to the front table. Finally, the subject released the object (RO) and retracted the arm (AR). The subject moved the object from the front table to the right table for the next cycle. The subject was informed about repeating these actions in 30 cycles. The decomposition of each action is illustrated in Figure 3A.

### 2.3. Data Acquisition System

The sensor used for detecting the EMG and FMG signal was developed by the author, and it was reported in the previous study (Ke et al., 2020). The co-located EMG-FMG sensor can measure the EMG signal and FMG signal at the same place simultaneously. In this study, five sensor modules were used for data acquisition. Therefore, 10 channels of signals (five channels EMG and five channels FMG) were collected. The five sensors were attached to an elastic cord with hook and loop fasteners and then wrapped surround the forearm (about 5 cm below the elbow joint). The sensors were not placed onto specific muscles precisely but were rather evenly distributed around the forearm. For the convenience of data processing, a National Instruments DAQ (NI-USB 6218) was used for data acquisition. The FSR channel attached to objects was also connected to the NI card. All of the channels were sampled at a frequency of 1,000 Hz. The structure of data acquisition is shown in Figure 3B.

### 2.4. Performance Verification of the Hardware Aspect

#### 2.4.1. Input Signal Source

Several classical pattern recognition models were used to compare the classification accuracy among three signal sources: EMG only, FMG only, and the combination of EMG and FMG.

#### 2.4.2. Feature Extraction

The raw EMG and FMG data were first filtered by a six-order bandpass Butterworth filter (the cutoff frequency is 20 and 450 Hz) to remove the movement artifact. Then the filtered signal of each channel was segmented separately by a sliding window for feature extraction. According to existing studies, the window length and step size significantly affect the accuracy and real-time latency. For real-time control, the latency time should be <300 ms (Zhang et al., 2019). To access the accuracy under different latency levels, we selected four latency times (the step size of the sliding window) for feature extraction: 50, 100, 150, and 200 ms. The length of the data window for feature extraction was twice the length of the step size, which means the sliding window had an overlap of 50%. For feature extraction of the EMG signal, many previous works indicated that the time-domain feature is powerful enough for classification. In this work, the feature set used for classification in this study includes four types of components, including the mean absolute values (MAV), root mean square (RMS), wave length (WL), and zero-crossing (ZC), the definition of each feature is referred to the description in Micera et al. (2010). Thus, for each window and each channel, four feature values were extracted. Therefore, the dimension of the feature vector of each signal mode (EMG or FMG, both have five channels) in a time window for classification is *R*^1 × 20^. Both the EMG and FMG signals were processed in the same way.

#### 2.4.3. Classification

Four commonly used classifiers were selected for comparison in this work, including the support vector machine (SVM) with linear kernel, the SVM with the second-order polynomial kernel, the linear discriminant analysis (LDA), and the k-nearest neighbors (kNN, the number of neighbors is set to be 50). The algorithm was implemented in MATLAB R2020b, and the classifiers we used here were directly called from the Matlab software. The features were normalized before inputting to the classifier, and the other parameters of these classifiers were kept in the software's default setting.

### 2.5. Performance Verification of the Algorithm Aspect

#### 2.5.1. Overview of the Algorithm

**Figure 4:**
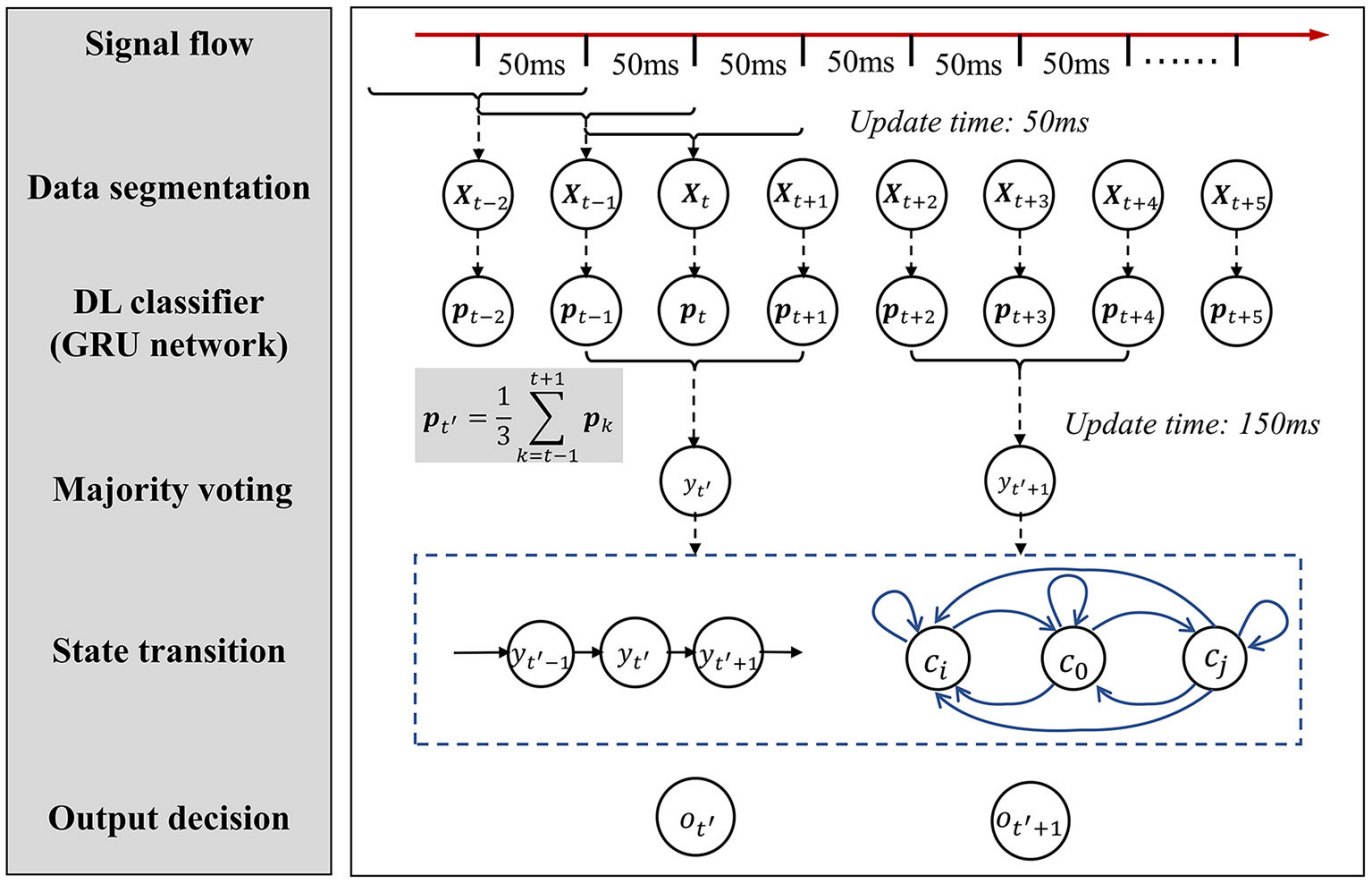
The pipeline flowchart of data processing, inference, and post decisions.

The algorithm consists of three stages: The first stage is an RNN-based model for fast inference (updates every 50 ms) based on the EMG and FMG signals. The output of this stage is the posterior probability of each grasp type rather than the discrete types; the second stage is a majority voting procedure (updates every 150 ms) based on the posterior probability of the first stage. This stage plays the role of smoothing the classification results to remove outliers; The third stage is an error correction model based on the state transition model summarized from the prior knowledge of the practical application. The pipeline of the algorithm is shown in Figure 4, details about each part are introduced below.

#### 2.5.2. Data Preparation

First, the raw EMG and FMG signals are filtered by a six-order bandpass Butterworth filter (the cutoff frequency is 20 and 450 Hz) to remove the artifact of movement. Then, the EMG and FMG signals (10 channels in total) are segmented into a window length of 100 ms. The data window has an overlap of 50 ms. Thus the update time (step size) of inference is 50 ms (suppose the time used for inferring is <50 ms). In this way, the dimension of the input signal to the next step is 10 × 100.

#### 2.5.3. Gated Recurrent Unit (GRU) Network

**Figure 5:**
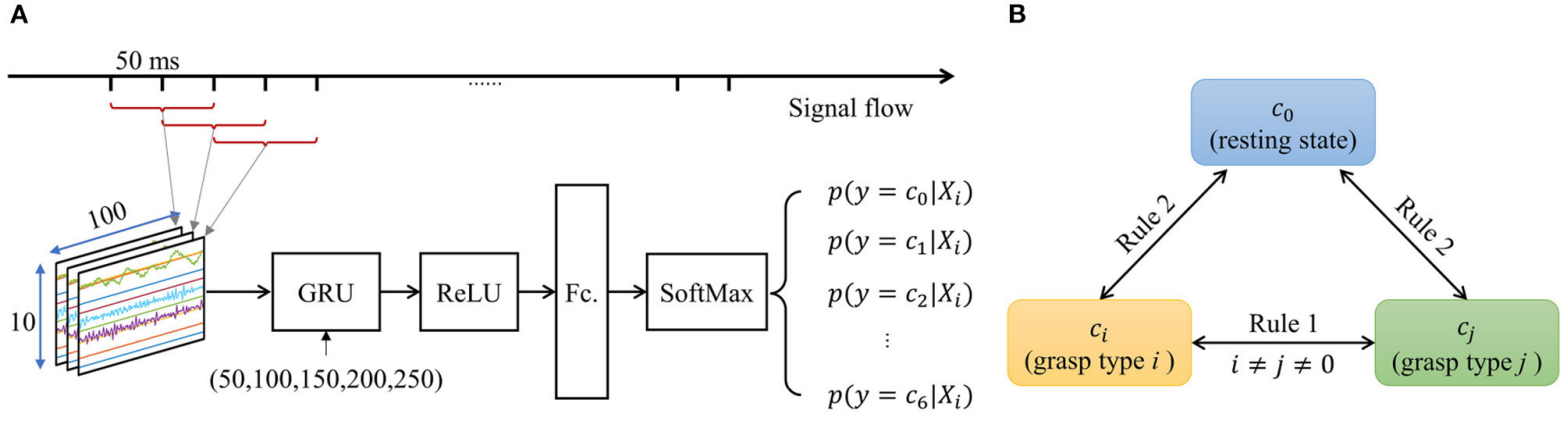
**(A)** The architecture of the GRU network. **(B)** The illustration of state transition.

A GRU network is adopted at the classification stage, which is a type of RNN. The architecture of the proposed GRU network is a simple combination of a sequential input layer, GRU layer, dropout layer, activation layer, fully connected layer, and output softmax layer, the architecture of the GRU network is shown in Figure 5A. The input signal matrix is normalized by a zero-score method at the sequential input layer. The number of hidden units in the GRU layer is a hyperparameter chosen from several numbers (100, 150, 200, 250, and 300) according to the average classification accuracy for all subjects. The activation layer we used here is the rectified linear unit (ReLU), which performs a threshold operation on each input value. Then the output from the ReLU layer is connected to a fully connected (Fc.) layer. Finally, a softmax layer is used for output. The output is set to be the posterior probability of each class.

The proposed architecture of the GRU network was implemented in MATLAB R2020b and trained using the adam optimizer. The batch size for training was set to be 128, and the number of training epochs was 30. The learning rate was set to be 0.001, while the factor for dropping the learning rate was 0.1. The other parameters were kept in the software's default setting. The hardware resource for the training process is a mobile workstation graphics card (Nvidia Quadro P620).

#### 2.5.4. Posterior Probability Smoothing

The update time of the GRU network was 50 ms, this time delay is too fast for prosthetic hand control, and it may cause many outlier commands. To obtain more reliable recognition results, we smoothed the posterior probability by averaging every three adjacent outputs of the GRU network: **P**_*t*−1_, **P**_*t*_, and **P**_*t*+1_. The time latency may increase to 150 ms. However, this latency is still under the constraint of real-time control.

The smoothed posterior probability for each step is given by:


(1) $$P_{t^{\prime }}=\frac{1}{3}\sum_{k=t-1}^{t+1}P_{k}$$

where ${\text{P}}_{t}={\left[p\left(y_{t}=c_{0}|{\text{X}}_{t}\right),p\left(y_{t}=c_{1}|{\text{X}}_{t}\right),...,p\left(y_{t}=c_{6}|{\text{X}}_{t}\right)\right]}^{T}$ is the output posterior probability of the softmax layer (*c*_0_ means rest, and *c*_1_ to *c*_6_ means the grasp type in Figures 2A–F). **X**_*t*_ is the input data matrix to GRU network. At this step, we calculate the output every 150 ms.

#### 2.5.5. State Transition Model for Decision

Considering the actual scenario, if the user keeps grasping an object, the next possible state will be keeping the current grasp type or releasing the object. It is impossible to change the grasp type from one to another directly without first releasing the current object in hand. Furthermore, from an empirical point of view, maintaining the current state may be greater than the probability of changing the state if safety and cost are considered. In other words, for a real-world prosthetic hand control system, the grasp type command of the last moment may affect the grasp type command of the next moment. For the PR-based control system, the grasp type command conversion conditions should be more strict on the posterior probability, rather than just choosing the grasp type with the highest posterior probability.

Based on the above analysis (prior knowledge), we summarized two simple decision-making rules based on the smoothed posterior probability. The output decision $o_{t^{\prime }}\in \left[c_{0},c_{1},...,c_{6}\right]$ at time *t*′ is determined by an iterative process.

**Rule 1**: the rule for transmitting from a grasp state *c*_*i*_, (*i*≠0) to the other grasp state *c*_*j*_, (*j*≠0, *j*≠*i*) is defined as:


(2) $$\begin{array}{c} if\;y_{t^{\prime }-1}\neq c_{0},y_{t^{\prime }}=c_{0}\;then\\ o_{t^{\prime }}=\{\begin{array}{c} o_{t^{\prime }-1}\frac{p(y_{t^{\prime }})}{p(y_{t^{\prime }-1})}<w_{2}\\ o_{t^{\prime }}\frac{p(y_{t^{\prime }})}{p(y_{t^{\prime }-1})}\geq w_{2} \end{array} \end{array}$$

where *w*_1_ is the threshold of state changing between different grasp types.

**Rule 2**: the rule for transmitting from a grasp state to the rest state is defined as:


(3) $$\begin{array}{c} if\;y_{t^{\prime }-1}\neq c_{0},y_{t^{\prime }}\neq c_{0},y_{t^{\prime }}\neq y_{t^{\prime }-1}\;then\\ o_{t^{\prime }}=\{\begin{array}{c} o_{t^{\prime }-1}\frac{p(y_{t^{\prime }})}{p(y_{t^{\prime }-1})}<w_{1}\\ o_{t^{\prime }}\frac{p(y_{t^{\prime }})}{p(y_{t^{\prime }-1})}\geq w_{1} \end{array} \end{array}$$

where *w*_2_ is the threshold of state changing from grasp to rest.

These two state transmission rules are described in Figure 5B. The value of *w*_1_ and *w*_2_ in rules 1 and 2 should be determined by the classifier's performance. They act as hyperparameters to adjust the strictness of state transition conditions. In this study, the value of *w*_1_ and *w*_2_ is optimized by directly searching from 1 to 5 with a span of 0.5. The final optimized value of *w*_1_ and *w*_1_ are both 2.5.

### 2.6. Training and Testing

To simulate the real-time training in a real-world application, we used the incremental training method for training. For each grasp type of each subject, the training data was the first 20 repetitions, and the remaining 10 were used for testing. This training strategy is similar to the real-world application since the training data should always precede the testing data in time (Zanghieri et al., 2019). For each subject and classifier (all of the evaluated classifiers in this work and different configurations of classifiers of the same type were regarded as different classifiers), the training and testing procedure was repeated 10 times to calculate the average accuracy. The average accuracy for all subjects is the mean value of each subject's average accuracy.

## 3. Results

### 3.1. Characteristics of EMG and FMG Signals

**Figure 6:**
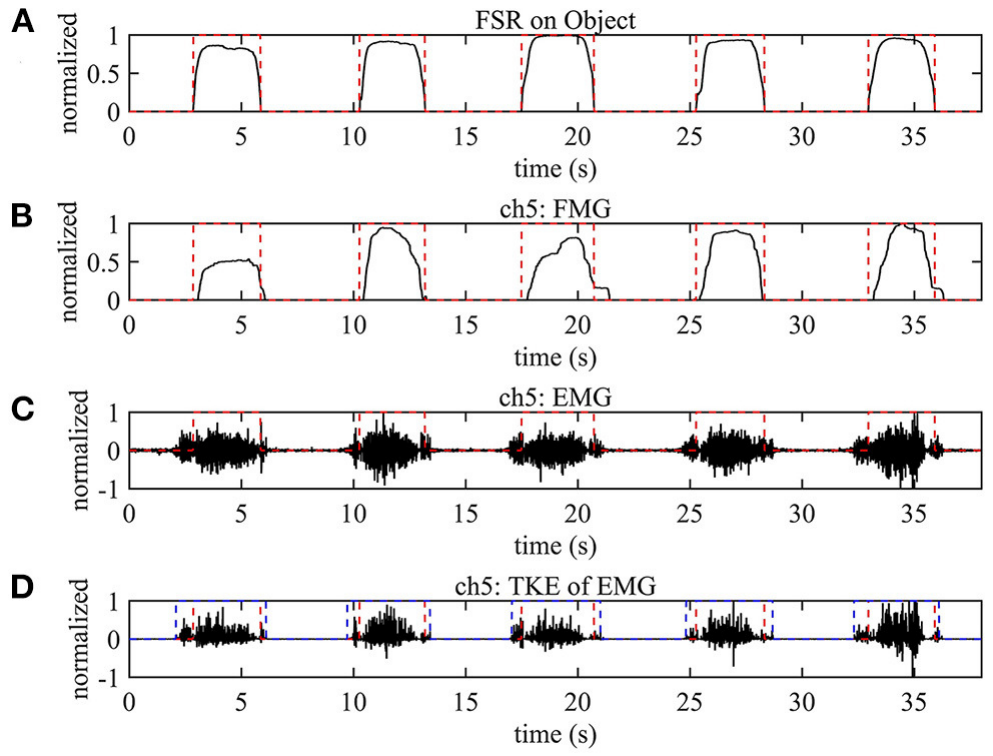
The example data of two channels during five consecutive reach-grasp-move task, the red dotted lines mean the true label of hand grasp or hand open (the change point of red dotted lines means the starting or ending of grasp) during five consecutive repetitions. **(A)** The trigger signal of FSR on objects. **(B)** The FMG signals on fifth channel. **(C)** The EMG signal on fifth channel. **(D)** The value of TKE operator of the EMG signals on fifth channel, the blue dotted line in denotes the onset and offset of the EMG signal.

Figure 6 shows the EMG and force signals of channel 5 in five consecutive grasp tasks (subject 1). The red dotted lines in Figures 6A–D mean the true label of hand grasp or hand open during five consecutive repetitions, the change point of red dotted lines means the starting or ending of grasp. The onset and offset of the grasping are determined by the force signal of the FSR sensor attached to the objects. The change point of red dotted lines was calculated by applying a hard threshold to the FSR signal in Figure 6A, the threshold is the mean value + 5 × standard deviation of the FSR signal in the first second (resting state). In comparison, the blue dotted line in Figure 6D denotes the onset and offset of the EMG signal in Figure 6C. The change point of the blue dotted line is determined from the Teager-Kaiser Energy (TKE) feature (Li et al., 2007) of the EMG signal by applying a hard threshold to the TKE feature. The threshold is the mean value + 5 × standard deviation of the TKE feature in the first second of the resting state. The TKE feature of the EMG signal is calculated by:


(4) $$\begin{array}{r} \psi_{i}={x_{i}}^{2}-x_{i-1}x_{i+1} \end{array}$$

where *x*_*i*_ is the EMG signal at time point *i*.

As shown in Figures 6C,D, the onset of the EMG signal is very sensitive to the arm movement. As long as the arm moves, there is an obvious EMG signal. This characteristic of the EMG signal is one of the reasons that cause the misclassification of the grasping gestures during dynamic arm movement. In contrast, as shown in Figure 6B, the FMG signal at the same position is not so sensitive to the arm movement. The onset of the FMG signal slightly lags behind the start of a steady grasp, which may be due to the mechanical low-frequency filtering effect of the FMG sensor. Generally speaking, the onset of the EMG signal is ahead of the onset of grasping, while the onset of the FMG signal lags behind the onset of grasping. Based on these phenomena, the combination of EMG and FMG might be a good choice for gesture recognition during dynamic arm movement.

### 3.2. EMG-FMG Signal Fusion for Classification

Table 1 shows the average accuracy in all subjects for different time windows and classifiers. For each subject, the accuracy is the mean value of 10 repeats, and then the accuracy of the eight subjects was averaged. It can be seen from Table 1 that the average accuracy of the EMG-FMG combination feature set is higher than that of only using EMG or FMG. For the traditional classifier, SVM still shows the best performance and robustness. Another fact should be noted is the influence of window length on accuracy. For linear SVM, a longer time window will get better performance when using EMG-only or FMG-only feature sets; However, the window length seems to have little influence on the EMG-FMG combined feature sets. Even though the time window is as short as 50 ms (which means an update frequency of inference is 20 Hz), the performance is still very steady. These facts also indicate that the combined EMG-FMG feature sets are more robust than each feature set.

**Table 1:** The average accuracy in all subjects for different time windows and classifiers.

**Classifier**	**Input signals**	**50 ms**	**100 ms**	**150 ms**	**200 ms**	**250 ms**
SVM (liner)	EMG	0.7749	0.7877	0.7990	0.8053	0.8054
	FMG	0.7724	0.8058	0.8181	0.8324	0.8350
	EMG-FMG	**0.8919**	**0.8984**	**0.8999**	**0.8974**	**0.8947**
SVM (second-order)	EMG	0.8170	0.8211	0.8139	0.8008	0.7964
	FMG	0.7894	0.7962	0.7990	0.8010	0.8014
	EMG-FMG	0.8805	0.8847	0.8893	0.8785	0.8734
LDA	EMG	0.6579	0.6874	0.7031	0.7153	0.7218
	FMG	0.6891	0.7245	0.7421	0.7560	0.7625
	EMG-FMG	0.8135	0.8388	0.8511	0.8557	0.8531
KNN	EMG	0.7498	0.7360	0.7211	0.7142	0.7038
	FMG	0.7143	0.7255	0.7259	0.7233	0.7115
	EMG-FMG	0.8064	0.7940	0.7802	0.7677	0.7531

*Bold values show the best results of classification (the maximum of accuracy)*.

**Figure 7:**
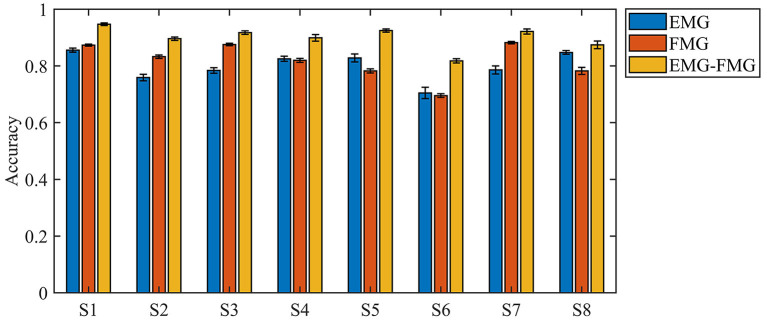
The accuracy of classification for three feature sets (classifier: linear SVM).

Among these classifiers, the SVM model with linear kernel function gets the best performance. Figure 7 shows the comparison of the accuracy of three kinds of feature sets for each subject when using the linear SVM classifier with a stepped window of 150 ms. The result shows that the accuracy of the combination of EMG-FMG features is significantly higher than the accuracy that only uses EMG or FMG (*p* < 0.05). However, the EMG and FMG features' accuracy does not show a consistent advantage for different subjects.

### 3.3. The Average Classification Accuracy

Table 2 shows the comparison of classification accuracy between our proposed model (the number of hidden units in the GRU layer is 150) and the other methods. Because no previous deep learning models used both EMG and FMG signals for input, the four deep learning models we mentioned here were all developed for EMG-only input. Among these four models, the model proposed by Nasri et al. (2019) was a GRU network, while the other three models were CNN networks with different structures (Atzori et al., 2016; Zia ur Rehman et al., 2018; Tam et al., 2019). Here, the FMG signal was treated the same way as the EMG signal for all models, which meant the input signals had 10 channels. The window length of input signals for all models was 300 ms, with an overlap of 150 ms. This way, the update time of decision outputs for all models was 150 ms.

**Table 2:** The comparison of average accuracy for different methods.

	**Atzori et al. (2016)**	**Zia ur Rehman et al. (2018)**	**Nasri et al. (2019)**	**Tam et al. (2019)**	**Linear SVM**	**This study**
Subject 1	0.8918	0.8575	0.7810	0.7568	0.9474	**0.9543**
Subject 2	0.8322	0.8181	0.7806	0.7034	0.8962	**0.9365**
Subject 3	0.9301	0.8797	0.8243	0.7654	0.9175	**0.9540**
Subject 4	0.8799	0.8360	0.7820	0.6997	0.8993	**0.9389**
Subject 5	0.8534	0.7970	0.7563	0.6772	0.9248	**0.9410**
Subject 6	0.8364	0.7894	0.7152	0.6525	0.8181	**0.9155**
Subject 7	0.9126	0.9071	0.8345	0.8244	0.9216	**0.9402**
Subject 8	0.8346	0.7860	0.6954	0.6625	0.8747	**0.9363**
Mean acc.	0.8714	0.8338	0.7712	0.7177	0.8999	**0.9396**

*Bold values show the best results of classification (the maximum of accuracy)*.

The results in Table 2 show that the performance of our proposed method is the best among these models. The accuracy of our proposed model is 3.5% higher than that of the SVM model. It also should be noted that the performance of the other four deep learning models is poorer than the classical SVM model. This result may be because the original paper of these models is all optimized for only EMG input. Moreover, the number of channels for this original research differs from our experiment. For example, the Atzori model is developed for the Ninapro database, which contains 12 channels and the number of EMG channels used in the work of Zia ur Rehman et al. (2018), Nasri et al. (2019), and Tam et al. (2019) were 8, 8, and 32, respectively. However, in this work, we used only five channels of EMG. The increasing of EMG channels may help improve the performance of the PR model.

### 3.4. The Role of Empirical Model

As we mentioned in Section 2.5.5, the empirical model in our method is focused on reducing the false-negative error. The false-negative error in this study is defined as a grasp type (*c*_1_ to *c*_6_) is misclassified as the rest type (*c*_0_). This kind of error should be more costly than a false-positive error.

**Figure 8:**
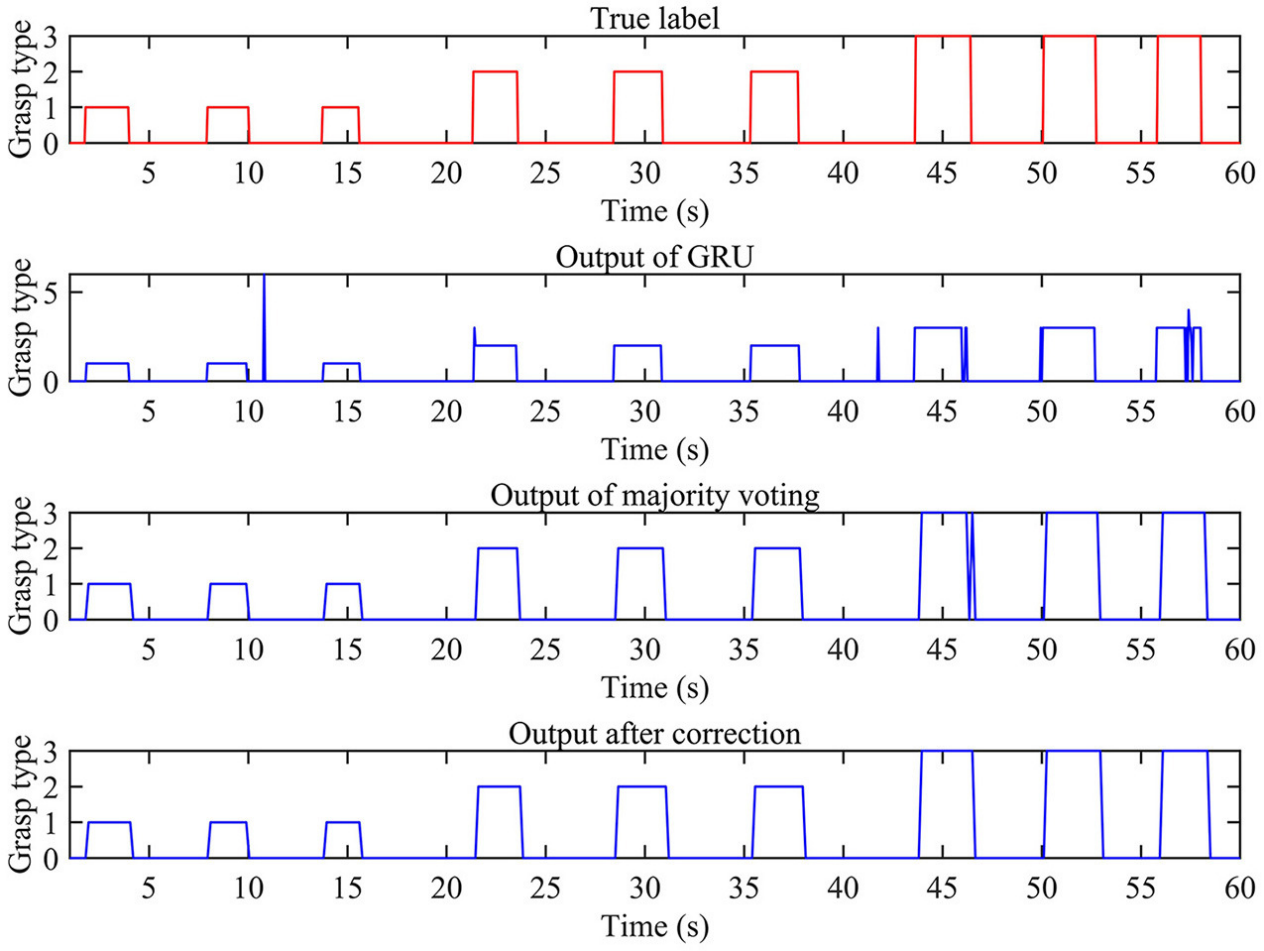
The continuous output of our proposed method at different stage.

Figure 8 shows the typical results of grasp gesture sequence over time before and after majority voting. The direct inference output of the GRU has many outliers, even though the general accuracy is still high enough. After the majority-voting method, the outliers are smoothed, but there is still some misclassification. However, this empirical model limits the variety of gestures, which may cause a lag behind the gesture change in mind.

**Figure 9:**
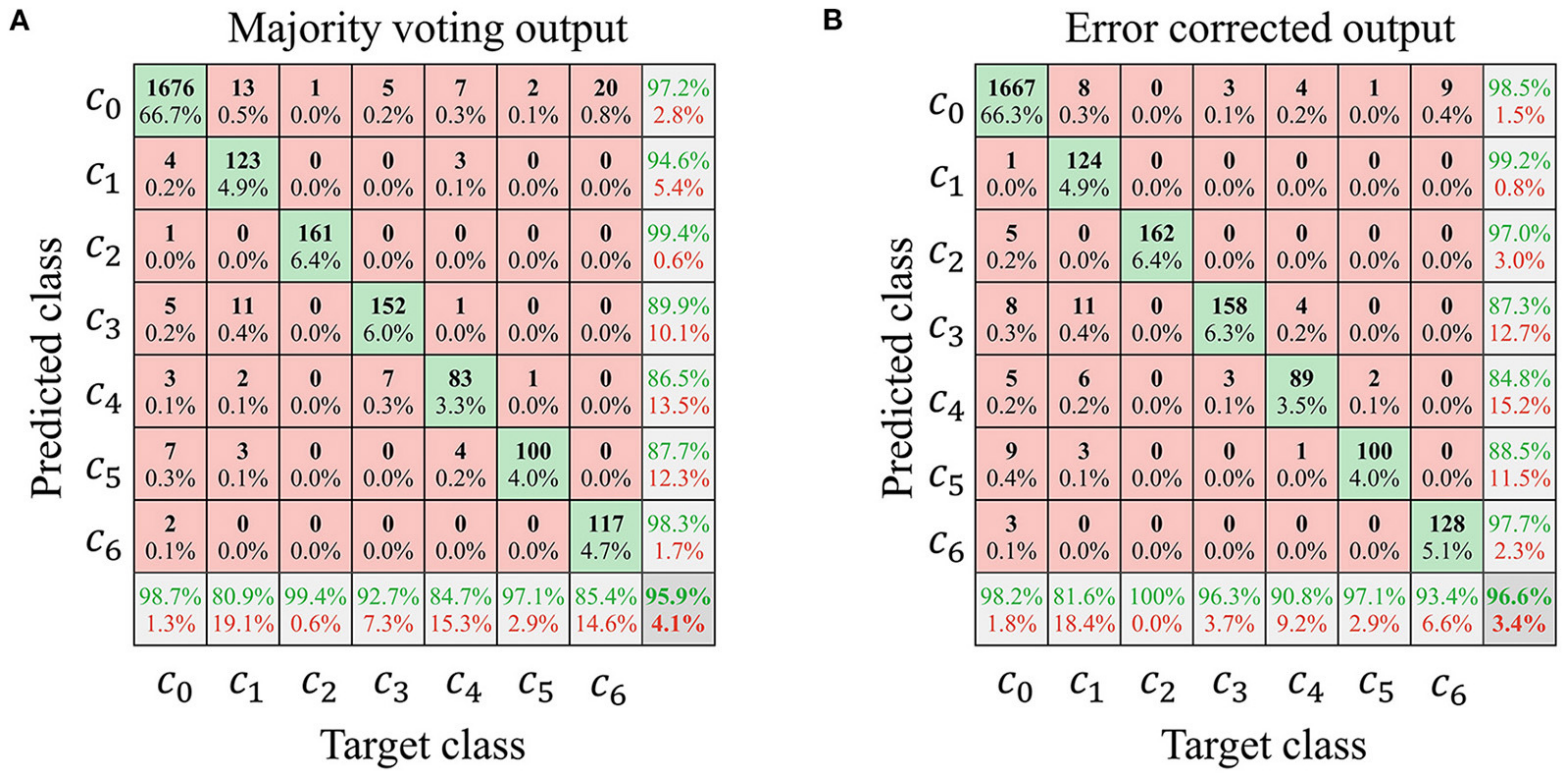
An example of the typical confusion matrix between **(A)** and after **(B)** error correction (subject 1).

Figure 9 is an example of the typical confusion matrix between and after error correction. The error correction model's role is to reduce the false-negative error that misclassified a grasp type into rest. Although the change in overall accuracy was not significant, the percentage that misclassified grasp as rest decreased, consistent with the purpose of the empirical model.

## 4. Discussion

This study provided a framework with great potential to reduce the interference of arm position change on PR-based prosthetic hand control. However, the study also has limitations: First, the data analysis is developed on a laptop rather than an embedded processor. On the embedded processor, the real-time performance (control latency) may be depressed due to the limitation of computation performance; In addition, the moving trajectory of the arm in the RGMRR task is relatively fixed, whereas, in real life, the trajectory of the arm may be random.

Whereas, the above is only reasonable speculation, the performance in the practical application needs further clinical online experimental research. In fact, in practical application, the user can intuitively observe the actual output results (according to the action and state of the prosthetic hand). Therefore, the user can adjust the intensity of grasping intention according to the actual output results. From this perspective, the result of real-time application may be better than off-line analysis because of the visual feedback in practical use.

## 5. Conclusion

For a PR system based on an EMG-only signal used for prosthesis control, the length of the signal window used for classification significantly influences classification accuracy. However, our study shows that the length of sliding window size has little effect on the PR system when using the co-located EMG and FMG signals as input signal sources. The performance of the PR system based on EMG-FMG signals input is better and more robust than EMG-only input or FMG-only input. The experimental results also show that the onset of the EMG signal is ahead of grasping, while the onset of the FMG signal lags behind grasping; thus, they may compensate for each other. These results indicate that the combination of EMG and FMG signals is a good multi-modal choice for classifying grasp types when arm position changes. Co-located EMG-FMG signals showed high robustness when arm position changes.

Compared with other direct classification methods, the algorithm proposed in this paper has two advantages: First, the average classification accuracy can be improved compared with other traditional methods or DL methods; Second, the false-negative error that misclassified a grasp type into rest can be controlled by adjusting the state transmission model, which provided a flexible way to balance the accuracy and action switching time.

Generally speaking, the experimental results showed that the proposed framework could improve the robustness of natural grasp type classification during arm movement. Thus, it may help narrow the academical-practical gap in PR-based myoelectric hand.

## Data Availability Statement

The raw data supporting the conclusions of this article will be made available by the authors, without undue reservation.

## Ethics Statement

The studies involving human participants were reviewed and approved by Medical Department of the People's Hospital in Yangxin, Hubei Province. The patients/participants provided their written informed consent to participate in this study.

## Author Contributions

AK participated in the development of proofs of concept under the supervision of JHu and JHe, and wrote the original draft. JHu and JW provided suggestions to improve the paper writing. All authors have read and agreed to the published version of the manuscript.

## Funding

This work was partially supported by the National Natural Science Foundation of China under Grant U1913207, by the Fund from Science, Technology, and Innovation Commission of Shenzhen Municipality (2021Szvup090), and by the Program for HUST Academic Frontier Youth Team.

## Conflict of Interest

The authors declare that the research was conducted in the absence of any commercial or financial relationships that could be construed as a potential conflict of interest.

## Publisher's Note

All claims expressed in this article are solely those of the authors and do not necessarily represent those of their affiliated organizations, or those of the publisher, the editors and the reviewers. Any product that may be evaluated in this article, or claim that may be made by its manufacturer, is not guaranteed or endorsed by the publisher.
